# Stochastic Selection of Activation Layers for Convolutional Neural Networks

**DOI:** 10.3390/s20061626

**Published:** 2020-03-14

**Authors:** Loris Nanni, Alessandra Lumini, Stefano Ghidoni, Gianluca Maguolo

**Affiliations:** 1 Department of Information Enginering, University of Padua, viale Gradenigo 6, 35131 Padua, Italy; stefano.ghidoni@unipd.it (S.G.); gianluca.maguolo@phd.unipd.it (G.M.); 2 DISI, Università di Bologna, Via dell’università 50, 47521 Cesena, Italy; alessandra.lumini@unibo.it

**Keywords:** Convolutional Neural Networks, ensemble of classifiers, activation functions, image classification, skin detection

## Abstract

In recent years, the field of deep learning has achieved considerable success in pattern recognition, image segmentation, and many other classification fields. There are many studies and practical applications of deep learning on images, video, or text classification. Activation functions play a crucial role in discriminative capabilities of the deep neural networks and the design of new “static” or “dynamic” activation functions is an active area of research. The main difference between “static” and “dynamic” functions is that the first class of activations considers all the neurons and layers as identical, while the second class learns parameters of the activation function independently for each layer or even each neuron. Although the “dynamic” activation functions perform better in some applications, the increased number of trainable parameters requires more computational time and can lead to overfitting. In this work, we propose a mixture of “static” and “dynamic” activation functions, which are stochastically selected at each layer. Our idea for model design is based on a method for changing some layers along the lines of different functional blocks of the best performing CNN models, with the aim of designing new models to be used as stand-alone networks or as a component of an ensemble. We propose to replace each activation layer of a CNN (usually a ReLU layer) by a different activation function stochastically drawn from a set of activation functions: in this way, the resulting CNN has a different set of activation function layers.

## 1. Introduction

Deep neural networks have become extremely popular as they achieve state-of-the-art performance on a variety of important applications including image classification, image segmentation, language processing, and computer vision [1]. Deep neural networks typically have a set of linear components whose parameters are usually learned to fit the data, and a set of nonlinearities, which are pre-specified, typically in the form of a sigmoid, a tanh function, a rectified linear unit, or a max-pooling function. The presence of nonlinear activation functions at each neuron is essential to give the network the ability of approximate arbitrarily complex functions [2], and its choice affects net accuracy and sometimes the speed of training. 

In this paper, we perform a large-scale empirical comparison of different activation functions across a variety of image classification and for an image segmentation problem. Starting from two of the best performing models, i.e. ResNet50 [3] for the classification task and DeepLabv3+ [4] for the segmentation task, we compare different approaches for replacing activation layers and different methods for building ensembles of CNNs obtained by varying the activation layers. 

After presenting and comparing several activation functions, we propose a new model based on the use of different activation functions at different levels of the graph: to this aim, we propose a method for stochastic selection of activation functions to replace each activation layer of the starting network. The activation functions are randomly selected from a set of nine approaches, including the most effective ones. After training the new models on the target problem, they are fused together to build an ensemble of CNNs. It is well known in the literature [5] that networks trained using back propagation are unstable; this behavior can be used for building an ensemble of classifiers. These networks are partially independent, and their fusion permits to boost the performance of a stand–alone network. 

The proposed framework for ensemble creation is evaluated on two different applications: image classification and image segmentation. In the image classification field, we deal with several medical problems by including in our benchmark 13 image classification datasets. Biomedical image retrieval is a challenging problem due to the varying contrast and size of structures in the images [6]. CNNs have already been used on several medical datasets reaching very high performance, including keratinocyte carcinomas and malignant melanomas detection [7], sub-cellular and stem cell image classification [8], thyroid nodules classification [9] from ultrasound images, or breast cancer recognition [10]. Our testing protocol includes a fine-tuning of each model in each dataset and a testing evaluation and comparison: our experiments show that the proposed ensembles work well in all the tested problems gaining state-of-the-art classification performance [11]. 

In the image segmentation field, we deal with the skin segmentation problem: the discrimination of skin and non-skin regions in a digital image has a wide range of applications including face detection [12], body tracking [13], gesture recognition [14], and objectionable content filtering [15]. Skin detection has great relevance also in the medical field, where it is employed as a component of face detection or body tracking: for example, in the remote photoplethysmography (rPPG) problem [16], it is a component of a system solving the problem of estimating the heart rate of a subject given a video stream of his/her face. In our experiment, we carry out a comparison of several approaches performing a single training on a small dataset including only 2000 labeled images, while testing is performed on 11 different datasets including images from very different applications. The reported results show that the proposed ensembles reach state-of-the-art performance [17] in most of the benchmark datasets even without ad-hoc tuning. 

The code developed for this work will be available at https://github.com/LorisNanni.

## 2. Literature Reviews

In the last years, deep learning has gained increasing attention in several computer vision applications, such as image classification and retrieval, object detection, image segmentation, and many other applications [18]. CNNs are deep neural networks designed to work similarly to the human brain in visual perception: CNNs are able to distinguish meaningful features in an image in order to classify the image as a whole. They are constituted of several types of layers of neurons: i.e., convolutional layers, activation layers, subsampling layers, and fully connected layers [19]. 

Most recent architectures present a substantially higher number of layers and parameters, which gives much more representation learning capability to those models. However, many parameters can produce overfitting. This problem can be solved with the introduction of regularization techniques, data augmentation, and better performing activation functions. 

In particular, the purpose of activation layers is to decide if a neuron would fire or not, according to a nonlinear transformation of the input signal. The design of new activation functions in order to improve training speed and network accuracy is an active area of research [20,21]. Recently, the sigmoid and hyperbolic tangent, which were the most widely used activations functions, have been replaced by Rectified Linear Units (ReLU) [22]: ReLU is a piecewise linear function equivalent to the identity for positive inputs and zero for negative ones. Thanks to the good performance of ReLU and the fact that it is fast, effective, and simple to evaluate, several alternatives to the standard ReLU function have been proposed in the literature. The most known “static” activation function are: Leaky ReLU [23], an activation function equal to ReLU for positive inputs but having a very small slope α > 0 for negative ones; ELU [21], which exponentially decreases to a limit point α in the negative space; and SELU [24], a scaled version of ELU (by a constant λ). Moreover, in [25], a randomized leaky rectified linear unit (RLReLU) is proposed, which uses nonlinear random coefficient instead of linear. The choice of optimal activation functions in a CNN is an important issue because it is directly related to the resulting success rates. Unfortunately, an analytical approach able to select optimal activation functions for a given problem is not available; therefore, several approaches try to determine them by trial and error. “Dynamic” activation functions are a class of function whose parameters, differently from “static” ones, are learned during training. Parametric ReLU (PReLU) [26] is a Leaky ReLU where the amount of the slope α is learned; Adaptive Piecewise Linear Unit (APLU) [20] is a piecewise linear activation function with learnable parameters: it calculates piecewise linear function independently for each neuron and learns them during the training process. Another “dynamic” function is proposed in [27], whose shape is learned by a linear regression model. In [28], two different variants are proposed: a “linear sigmoidal activation”, which is a fixed structure function whose function coefficients are static, and its “dynamic” variant, named “adaptive linear sigmoidal activation”, which can adapt itself according to the complexity of the given data. Two of the best performing functions are Swish [29], which is the combination of a sigmoid function and a trainable parameter, and the recent Mexican ReLU (MeLU) [30], which is a piecewise linear activation function that is the sum of PReLU and multiple Mexican hat functions. 

The main difference between “static” and “dynamic” functions is that the first class of activations considers all the neurons and layers as identical, while second class learns parameters independently for each layer or even each neuron. Although the “dynamic” activation functions perform better than “static” in some applications, their parametric nature increases the number of trainable parameters and thus the possibility of overfitting. In this work, we propose a mixture of “static” and “dynamic” activation functions. 

## 3. Activation Functions

This study considers 10 different activation functions (more details, and specific reference for each function, are given in [30]), namely the widely used ReLU and several variants. The functions used are summarized in Table 1, while in the following the analytical expression together with their derivatives are given. Several dynamic activation functions depend on a hyperparameter, named maxInput, which is a normalization factor to better deal with input images varying between [0,1] or [0,255].

The well-known ReLU activation function, for the generic couple of points $\left(x_{i},\;y_{i}\right)$, is defined as:

(1) $$y_{i}=f\left(x_{i}\right)=\{\begin{array}{cc} 0,\; & x_{i}<0\\ x_{i},\; & x_{i}\geq 0 \end{array}$$

and its derivative is easily evaluated as:

(2) $$\frac{dy_{i}}{dx_{i}}=f\prime \left(x_{i}\right)=\{\begin{array}{cc} 0,\; & x_{i}<0\\ 1,\; & x_{i}\geq 0 \end{array}$$

This work also considers several variants of the original ReLU function. The first variant is the Leaky ReLU function, defined as:

(3) $$y_{i}=f\left(x_{i}\right)=\{\begin{array}{r} ax_{i},\;\;x_{i}<0\\ x_{i},\;\;x_{i}\geq 0 \end{array}$$

where the parameter a is a small real number (0.01 in this study). The main advantage of Leaky ReLU is that the gradient is always positive (no point has a zero gradient):

(4) $$\frac{dy_{i}}{dx_{i}}=f\prime \left(x_{i}\right)=\{\begin{array}{r} a,\;\;x_{i}<0\\ 1,\;\;x_{i}\geq 0 \end{array}$$

The second variant of the ReLU function considered in this work is the Exponential Linear Unit (ELU) [21], which is defined as:

(5) $$y_{i}=f\left(x_{i}\right)=\{\begin{array}{r} a\left(\exp x_{i}-1\right),\;\;x_{i}<0\\ x_{i},\;\;x_{i}\geq 0 \end{array}$$

where a is a real number (1 in this study). ELU has a gradient that is always positive:

(6) $$\frac{dy_{i}}{dx_{i}}=f\prime \left(x_{i}\right)=\{\begin{array}{r} a\;\exp\left(x_{i}\right),\;\;x_{i}<0\\ 1,\;\;x_{i}\geq 0 \end{array}$$

The Parametric ReLU (PReLU) is the third variant that is considered here. It is defined by:

(7) $$y_{i}=f\left(x_{i}\right)=\{\begin{array}{r} a_{c}x_{i},\;\;x_{i}<0\\ x_{i},\;\;x_{i}\geq 0 \end{array}$$

where $a_{c}$ is a set of real numbers, one for each input channel. PReLU is similar to Leaky ReLU, the only difference being that the $a_{c}$ parameters are learned. The gradient of PReLU is:

(8) $$\frac{dy_{i}}{dx_{i}}=f\prime \left(x_{i}\right)=\{\begin{array}{r} a_{c},\;\;x_{i}<0\\ 1,\;\;x_{i}\geq 0 \end{array}\;\mathrm{and}\;\frac{dy_{i}}{da_{c}}=\{\begin{array}{r} x_{i},\;\;x_{i}<0\\ 0,\;\;x_{i}\geq 0 \end{array}$$

S-Shaped ReLU (SReLU) is the fourth variant. It is defined as a piecewise linear function:

(9) $$y_{i}=f\left(x_{i}\right)=\{\begin{array}{cc} t^{l}+a_{\;}^{l}\left(x_{i}-t^{l}\right),\;\; & x_{i}<t^{l}\\ x_{i},\;\; & t^{l}\leq x_{i}\leq t^{r}\\ t^{r}+a_{\;}^{r}\left(x_{i}-t^{r}\right),\;\; & x_{i}>t^{r} \end{array}$$

In this case, four learnable parameters are used, $t^{l},t^{r},a^{l},$ and $a^{r}$, expressed as real numbers. They are initialized to $a^{l}=0,\;t^{l}=0$, $a^{r}=1$, and $t^{r}=maxInput$. SReLU is highly flexible thanks to the rather large number of tunable parameters. The gradients are given by:

(10) $$\frac{dy_{i}}{dx_{i}}=f\prime \left(x_{i}\right)=\{\begin{array}{cc} a_{\;}^{l},\;\; & x_{i}<t^{l}\\ 1,\;\; & t^{l}\leq x_{i}\leq t^{r}\\ a_{\;}^{r},\;\; & x_{i}>t^{r} \end{array}$$

(11) $$\frac{dy_{i}}{da^{l}}=\{\begin{array}{r} x_{i}-t^{l},\;\;x_{i}<t^{l}\\ 0,\;\;x_{i}\geq t^{l} \end{array},\;\mathrm{and}$$

(12) $$\frac{dy_{i}}{dt^{l}}=\{\begin{array}{r} -a^{l},\;\;x_{i}<t^{l}\\ 0,\;\;x_{i}\geq t^{l} \end{array}$$

The fifth variant is APLU (Adaptive Piecewise Linear Unit). As the name suggests, it is a linear piecewise function. It is defined as:

(13) $$y_{i}=\mathrm{ReLU}\left(x_{i}\right)+\overset{n}{\underset{c=1}{\sum }}a_{c}\min\left(0,-x_{i}+b_{c}\right)$$

where *n* is an hyperparameter, set in advance, defining the number of functions (or hinges); and $a_{c}$ and $b_{c}$ are real numbers, one for each input channel. The gradients are evaluated as:

(14) $$\frac{df\left(x,a\right)}{da_{c}}=\{\begin{array}{r} -x+b_{c},\;\;x<b_{c}\\ 0,\;\;x\geq b_{c} \end{array}\;\;\mathrm{and}\;\frac{df\left(x,a\right)}{db_{c}}=\{\begin{array}{c} -a_{c},\;\;x<b_{c}\\ 0,\;\;x\geq b_{c} \end{array}\;$$

In our tests, the parameters $a_{c}$ are initialized to 0, and the points are randomly chosen. We also added an $L^{2}$-penalty of 0.001 to the norm of the parameters $a_{c}$.

An interesting variant is the Mexican ReLU (MeLU), derived from the Mexican hat functions. These are defined as:

(15) $$\phi^{a,\;\lambda }\left(x\right)=\max\left(\lambda \cdot maxInput-\left|x-a\cdot maxInput\right|,0\right)$$

where a and λ are real numbers. These functions are used to define the MeLU function, based on the definition of the PReLU detailed above:

(16) $$y_{i}=\mathrm{MeLU}\left(x_{i}\right)={\mathrm{PReLU}}^{c_{0}}\left(x_{i}\right)+\sum_{j=1}^{k-1}c_{j}\;\phi^{\alpha_{j},\lambda_{j}}\left(x_{i}\right)$$

The parameter k represents the number of learnable parameters for each input channel, $c_{j}$ are the learnable parameters, $c_{0}$ is the parameter vector in PReLU, and $\alpha_{j}$ and $\lambda_{j}$ are fixed parameters chosen recursively. The MeLU activation function has interesting properties, inherited from the Mexican hat functions, that are continuous and piecewise differentiable. ReLU can be seen as a special case of MeLU, when all the $c_{i}$ parameters are set to 0. This is important because pre-trained networks based on the ReLU function can be enhanced in a simple way using MeLU. Similar substitutions can be made when the source network is based on Leaky ReLU and PReLU.

As previously observed, MeLU is based on a set of learnable parameters. The number of parameters is sensibly higher with respect to SReLU and APLU, making MeLU more adaptable and with a higher representation power but more likely to overfit. The gradient is given by the Mexican hat functions. The MeLU activation function also has a positive impact on the optimization stage.

In our work, the learnable parameters are initialized to 0, meaning that the MeLU starts as a plain ReLU function; the peculiar properties of the MeLU function come into play at a later stage of the training. The first Mexican hat function has its maximum in 2·maxInput and is equal to zero in 0 and 4·maxInput. The next two functions are chosen to be zero outside the interval [0, 2·maxInput] and [2·maxInput, 4·maxInput], with the requirement being they have their maximum in maxInput and 3·maxInput. The parameters α and λ are chosen to fulfill this requirement. 

In this work we test two values of *k*, the standard value is *k* = 4 for MeLU and a wider version of the function for *k* = 8 (wMeLU). 

The Gaussian ReLU, also called GaLU, is the last activation function considered in our work. Its definition is based on the Gaussian type functions:

(17) $$\phi_{g}{}^{a,\;\lambda }\left(x\right)=\max\left(\lambda \cdot maxInput-\left|x-a\cdot maxInput\right|,0\right)+\;+\min\;(\left|x-a\cdot maxInput-2\lambda \cdot maxInput\right|-\lambda \cdot maxInput,0)$$

where a and λ are real numbers. The GaLU activation function is defined as:

(18) $$y_{i}=GaLU\left(x_{i}\right)=PReLU^{c_{0}}\left(x_{i}\right)+\sum_{j=1}^{k-1}c_{j}\;\phi_{g}{}^{a_{j},\lambda_{j}}\left(x_{i}\right)$$

which is a formulation similar to the one provided for MeLU, which again depends on the parameters $a_{j}$ and $\lambda_{j}$. Again, the function is defined in this way to provide a good approximation of nonlinear functions. We use k=4 for GaLU and k=2 for its “smaller” version sGaLU.

Please note that, to avoid any overfitting, we use the same parameter setting suggested by the original authors for each activation function, as reported in Table 1.

## 4. Materials and Methods

In this section, we describe both the starting models and the stochastic method proposed to design new CNN models and create ensembles. In the literature, several CNN architectures have been proposed for image classification (AlexNet [32], GoogleNet [33], InceptionV3 [34], VGGNet [35], ResNet [3], and DenseNet [36]) and segmentation problems (SegNet [37], U-Net [38], and Deeplabv3+ [4]). In our experiments, we selected two of the best performing models: ResNet50 [3] for image classification and Deeplabv3+ [4] for segmentation. ResNet50 is a 50-layer network, which introduces a new “network-in-network” architecture using residual layers. ResNet50, which was the winner of ILSVRC 2015, is one of the best performing and most popular architectures used for image classification. In our experiments, all the models for image classification were fine-tuned on the training set of each classification problem according to the model training parameters reported in Table 2. Data augmentation includes random reflection on both axes and two independent random rescales of both axes by two factors uniformly sampled in [1,2]. 

For image segmentation purposes, we selected Deeplabv3+ [4], a recent architecture based on atrous convolution, in which the filter is not applied to all adjacent pixels of an image but rather to a spaced-out lattice of pixels. Deeplabv3+ uses four parallel atrous convolutions (each with differing atrous rates) followed by a “Pyramid Pooling” method. Since DeepLabv3+ is based on encoder–decoder structure, and it can be built on top of a powerful pre-trained CNN architecture: in this work, we selected again ResNet50 for this task, although our internal evaluation showed that ResNet101 and ResNet34 gained similar performance. All the models for skin segmentation were trained on a small dataset of 2000 images using class weighting and the same training parameters, as reported in Table 2.

Given a base model for each task, i.e. ResNet50 for image classification and DeepLabv3+ for skin segmentation, we designed several variants of the initial architecture by replacing all the activation layers (which were ReLU layers in both the starting models used in this work) by a different activation function. The stand-alone methods named *leakyReLU, ELU, SReLU, APLU, GaLU, sGaLU, PReLU, MeLU,* and *wMeLU* together with the original model (*ReLU*) are the 10 models tested in our experiments. Some of them depend on the training parameter maxInput, which was set to 1 if not specified (255, otherwise).

**Figure 1:**
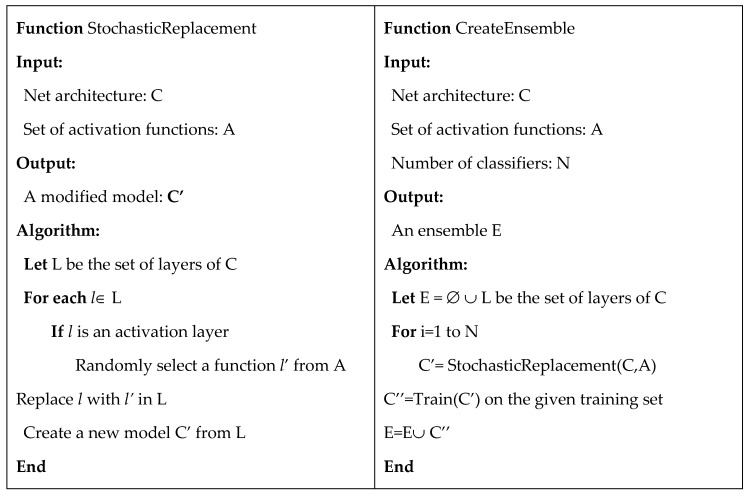
Pseudo-code of the two procedures for stand-alone random model and ensemble creation.

After comparing several activation functions, we propose to design a new model based on the use of different activation functions in different layers. According to the pseudo-code in Figure 1, a *RandAct* model is obtained using the function StochasticReplacement, applied to an input CNN and a set of activation functions, by randomly replacing all the activation layers of the input model. In our experiments, we considered ResNet50 as the input model for image classification and DeepLabv3+ for image segmentation. However, this method is general and it could be applied to any other model. The output models *RandAct* and *RandAct*(255) were obtained from input models using the set of 9 alternative activation functions with the maxInput parameter equal to 1 or 255. To create an ensemble, the function CreateEnsemble is used: first, StochasticReplacement is used to generate N *RandAct* models, then the models are fine-tuned on the training set, and finally they were fused together in an ensemble using the sum rule. The fusion of CNNs using the sum rule consists in summing the outputs of the last softmax layer. Then, the final decision is obtained applying an argmax function. In the segmentation task, we evaluated the sum of the output mask, which is equal to a vote rule at pixel level. The ensemble created and tested in the experimental section are the following:

- *FusRan10* and *FusRan10*(255) are ensembles obtained by the fusion of 10 *RandAct* or *RandAct* (255) models (i.e., fixing maxInput = 1 or 255)
- *FusRan20* = *FusRan10* + *FusRan10*(255)
- *FusRan3* and *FusRan3*(255) are the ensembles obtained by the fusion of 3 stochastic models as *RandAct* or *RandAct*(255).

Moreover, we also tested the following ensembles obtained by the sum rule of the above stand-alone models:

- *FusAct10* and *FusAct10*(255) are the ensembles obtained by the fusion of all the 10 non-random stand-alone models obtained by varying the activation functions: i.e. *ReLU*, *leakyReLU, ELU, SReLU, APLU, GaLU, sGaLU, PReLU, MeLU,* and *wMeLU* (fixing maxInput to 1 or 255)
- *FusAct3* is a lightweight ensemble obtained by the fusion of the best 3 stand-alone models (evaluated on the training set), *FusAct3* = *wMeLU* + *MeLU* + *PReLU* for skin classification
- *FusAct3*(255) is a lightweight ensemble obtained by the fusion of the best 3 stand-alone methods for image classification, *FusAct3(255)* = *wMeLu(255)* + *MeLu(255)* + *SReLu(255)*

Finally, we proposed two ensembles obtained mixing different types of selection for activation functions:

- *FusAR20* = *FusAct10* + *FusRan10*
- *FusAR20*(255) = *FusAct10*(255) + *FusRan10*(255)

## 5. Results

To evaluate the stand-alone models based on different activation functions, the stochastic method for model and ensemble creation and the other ensembles described in Section 4, we performed experiments on 13 well-known medical datasets for image classification and 11 datasets for skin segmentation. Table 3 summarizes the 13 datasets for image classification including a short abbreviation, the dataset name, the number of samples and classes, the size of the images, and the testing protocol. We used five-fold cross-validation (5CV) in 12 out of 13 datasets, while we maintained a three-fold division for the Laryngeal dataset (the same protocol in [39]). Table 4 summarizes the 11 datasets used for skin segmentation. All models were trained only on the first 2000 images of the ECU dataset [40]; therefore, the other skin datasets were used only for testing (for ECU, only the last 2000 images not included in the training set were used for testing).

The evaluation and comparison of the proposed approaches was performed according to two of the most used performance indicators in image classification and skin segmentation: accuracy and F_1_-measure, respectively. Accuracy is the ratio between the number of true predictions and the total number of samples, while the F_1_-measure is the harmonic mean of precision and recall and it is calculated according to the following formula F_1_ =2tp/(2tp+fn+fp), where *tn, fn, tp,* and *fp* are the number of true negatives, false negatives, true positives, and false positives evaluated at pixel-level, respectively. According to other works on skin detection, F_1_ was calculated at pixel-level (and not at image-level) to be independent on the image size in the different databases. Finally, to validate the experiments, the Wilcoxon signed rank test [56] was used. For our experiments, all images were resized to the input size of the CNN models (i.e., 224 × 224 for ResNet50 and all our variants) before training and testing, and then the output mask for skin segmentation was resized back to original size. 

In the first experiment, we evaluated the proposed methods for image classification on the datasets listed in Table 3. Table 5 reports the accuracy obtained by all the tested stand-alone models and ensembles: the last two columns report the average accuracy (Avg) and the rank (evaluated on Avg). 

From the results in Table 5, we can draw the following conclusions:

- All ensembles are ranked before the stand-alone methods: this demonstrates that changing the activation function is a viable method for creating diversity among models.
- The method named *ReLU*, which is our baseline since it is the standard implementation of ResNet50, performs very well, but it is not the best performing activation function: many activation functions (with the maxInput = 255) perform better than *ReLU* on average.
- It is a very valuable result that methods such as *wMeLU*(255), *MeLU*(255), and some other stand-alone approaches strongly outperform *ReLU*. Starting from a pretrained model and changing its activation layers, we obtained a sensible error reduction. This means that our approaches permit boosting the performance of the original ResNet50 on a large set of problems.
- It is difficult to select a function that wins in all problems. Therefore, a good method to improve performance is to create an ensemble of different models: both *FusAct10* and *FusAct10*(255) work better than each of their single components.
- Designing the models by means of stochastic activation functions (i.e., *RandAct* or *RandAct*(255)) gives valuable results: *RandAct* is ranked 12th, only two positions worse than the best stand-alone model (*wMeLU*(255) ranked 10th) and before the baseline *ReLU* (15th).
- Moreover, the selection of stochastic activation functions is very valuable for the creation of ensembles: both *FusRan10* and *FusRan10*(255) perform very well compared to all stand-alone models and other ensembles; their fusion *FusRan20 = FusRan10* + *FusRan10*(255) is the first ranked method tested on these experiments.
- The two small ensembles *FusAct3*(255) and *FusRan3*(255) perform very well; they strongly outperform stand-alone approaches and reach performance comparable with other heavier ensembles (composed of 10 or 20 models).

In the second experiment, we evaluated the proposed methods for skin segmentation on the 11 datasets listed in Table 4. In Table 6, the performance of all the tested stand-alone models and ensembles are reported in terms of F_1_-measure; the last two columns report the average F_1_-measure (Avg) and the rank (calculated on the average F_1_).

From the results in Table 6 it can be derived that:

- *ReLU* is the standard DeepLabv3+ segmentation CNN based on ResNet50 encoder. This is our baseline, since it has shown state-of-the-art performance for skin segmentation [17]. Many stand-alone models based on different activation functions outperform *ReLU:* in this problem, the activation functions with maxInput = 1 work better than those initialized at 255; therefore, we set to 1 the maxInput for the ensembles with three models (FusAct3 and FusRan3).
- Similar to the image classification experiment, all ensembles work better than any stand-alone approach: *FusAR20* is the best ranked method in our experiments, but two “lighter” ensembles, namely *FusAct3* and *FusAct10*, offer very good performance.
- Similar to the classification problem, the proposed approaches outperform *ReLU*, i.e. the standard DeepLabv3+ based on ResNet50, a state-of-the-art approach for image segmentation.
- The reported results show that all the proposed ensembles reach state of the art performance [17] in most of the benchmark datasets: all of them outperform our baseline *ReLU*.

**Figure 2:**
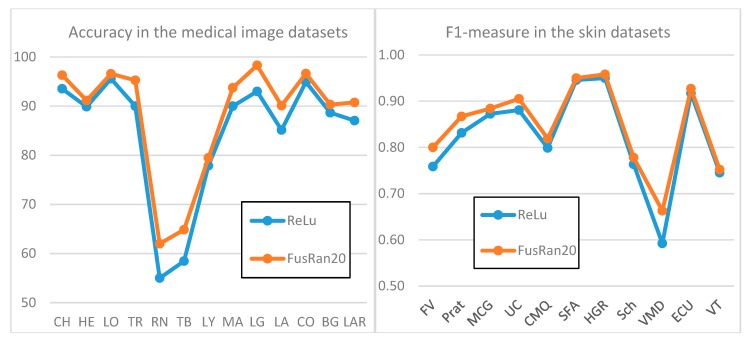
Comparison among the baseline *ReLU* and the ensemble *FusRan20* in both the problems.

**Figure 3:**
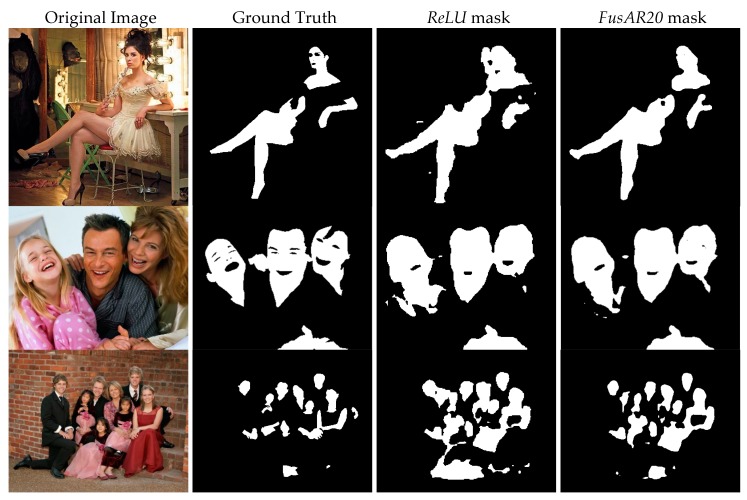
Visual comparison of segmentation results on images from Pratheepan dataset (from left to right): original input image, ground truth, *ReLU* output mask, and *FusAR20* output mask.

To give a visual evidence of the performance improvement obtained by our ensemble *FusRan20* with respect to the baseline *ReLU,* Figure 2 presents two graphics of performance on both datasets. Moreover, in Figure 3, sample output masks from the Pratheepan dataset obtained by our ensemble *FusAR20* with respect to the baseline *ReLU* and the ground truth are shown. In all three sample images, the improvement of the ensemble with respect to our baseline stand-alone method is clearly visible. 

Finally, we report some comparisons considering the Wilcoxon signed rank test. In Table 7 and Table 8, we compare the performance of some approaches for classification and segmentation: we selected the most interesting approach for each size of ensembles (of course, the approaches can be different in the two problems). The reported p-values confirm the conclusions drawn from Table 5 and Table 6. Moreover, the Wilcoxon signed rank test between *FusRan10* and *FusAct10* shows that the stochastic ensemble outperforms the other one with a p-value of 0.0166 on our 13 datasets for image classification. Similarly, *FusRan10*(255) outperforms *FusAct10*(255) with a p-value of 0.0713 in the image classification problem. This is an experimental demonstration that introducing a stochastic selection is a method to improve diversity of classifiers.

Finally, using a Titan Xp, the classification time of a ResNet50 is 0.018 s per image; this mean that, using an ensemble of 20 CNNs, it is possible to classify more than two images per second using a single Titan Xp. 

## 6. Conclusions

In this study, we proposed a method for CNN model design based on changing all the activation layers of the best performing CNN models by stochastic layer replacement. We proposed to replace each activation layer of a CNN by a different activation function stochastically drawn from a given set. This means that the resulting model has different activation function layers. This generation process introduces diversity among models making them suitable for ensemble creation. Interestingly, this design approach has gained very strong performance for ensemble creation: a set of ResNet50-like models designed using stochastic replacement of all activation layers and combined by sum rule strongly outperforms both standard ResNet50 (i.e., with a static *ReLU* activation function) and a single stochastic model (i.e., *RandAct*) in our experiments. A large experimental evaluation was carried out on a wide set of benchmark problems both for image classification and image segmentation. Experimental results demonstrate that the proposed idea is very effective to build a high-performance ensemble of CNNs. 

Even if these first results are limited to a single, albeit highly performing model, we plan as a future work to assess the proposed method on a larger class of models including lighter architectures suitable for mobile devices. The difficulty of studying ensembles of CNNs lies in the enormous speed and memory resources required to conduct such experiments. 

## Figures and Tables

**Table 1:** Summary of the activation functions evaluated in this work: name, parameter settings or initialization (if they are learned), learned parameters, and reference.

Name	Parameters Setting/Initialization	Learned Parameters	Ref
ReLu	--	--	[22]
Leaky ReLU	a = 0.1	--	[23]
ELU	a=1	--	[21]
PReLU	$a_{c}\;$= 0	$a_{c}$	[26]
SReLU	$a^{l}=0,\;t^{l}=0$, $a^{r}=1$$t^{r}=maxInput$	$a^{l},t^{l},a^{r},t^{r}$	[31]
APLU	*N* = 3, $a_{c}=0$,$\;b_{c}=rand()*maxInput$,	$a_{c},b_{c}$	[20]
MeLU	K=4, α=[2,1,3], λ=[2,1,1]	$c\in R^{k}$	[30]
wMeLU	K=8, α=[2,1,3,0.5,1.5,2.5,3.5], λ=[2,1,1,0.5,0.5,0.5,0.5]	$c\in R^{k}$	[30]
GaLU	K = 4, α=[1,0.5,2.5], λ=[1,0.5,0.5]	$c\in R^{k}$	
sGaLU	K = 2, α=[1], λ=[1]	$c\in R^{k}$	

**Table 2:** Model training parameters used for image classification and skin segmentation.

Parameter	Image Classification	Skin Segmentation
batch size	32	32
learning rate	0.0001	0.001
max epoch	30	50
data augmentation	Yes	yes (30 epoch)

**Table 3:** Summary of the Medical Datasets for image classification: short same (ShortN), name, number of classes (#C), number of samples (#S), image size, testing protocol, and reference.

ShortN	Name	#C	#S	Image Size	Protocol	Ref
CH	Chinese hamster ovary cells	5	327	512 × 382	5CV	[41]
HE	2D HELA	10	862	512 × 382	5CV	[41]
LO	Locate Endogenous	10	502	768 × 512	5CV	[42]
TR	Locate Transfected	11	553	768 × 512	5CV	[42]
RN	Fly Cell	10	200	1024 × 1024	5CV	[43]
TB	Terminal bulb aging	7	970	768 × 512	5CV	[43]
LY	Lymphoma	3	375	1388 × 1040	5CV	[43]
MA	Muscle aging	4	237	1600 × 1200	5CV	[43]
LG	Liver gender	2	265	1388 × 1040	5CV	[43]
LA	Liver aging	4	529	1388 × 1040	5CV	[43]
CO	Human colorectal cancer	8	5000	150 × 150	5CV	[44]
BGR	Breast grading carcinoma	3	300	1280 × 960	5CV	[45]
LAR	Laryngeal dataset	4	1320	1280 × 960	Tr-Te	[39]

**Table 4:** Summary of the Skin detection datasets with ground truth used for image segmentation: short name (ShortN), name, number of images (#S), quality of the ground truth, and reference.

ShortN	Name	#S	Ground Truth	Ref
CMQ	Compaq	4675	Semi-supervised	[46]
UC	UChile DB-skin	103	Medium Precision	[47]
ECU	ECU Face and Skin Detection	4000	Precise	[40]
Sch	Schmugge dataset	845	Precise (3 classes)	[48]
FV	Feeval Skin video DB	8991	Low quality, imprecise	[49]
MCG	MCG-skin	1000	Imprecise	[50]
Prat	Pratheepan	78	Precise	[51]
VMD	5 datasets for human activity recognition	285	Precise	[52]
SFA	SFA	1118	Medium Precision	[53]
HGR	Hand Gesture Recognition	1558	Precise	[54]
VT	VT-AAST	66	Precise	[55]

**Table 5:** Performance of the proposed approaches in the medical image datasets (accuracy).

	Dataset													Avg	Rank
Method	CH	HE	LO	TR	RN	TB	LY	MA	LG	LA	CO	BG	LAR		
ReLU	93.5	89.9	95.6	90.0	55.0	58.5	77.9	90.0	93.0	85.1	94.9	88.7	87.1	84.55	15
leakyReLU	89.2	87.1	92.8	84.2	34.0	57.1	70.9	79.2	93.7	82.5	95.7	90.3	87.3	80.30	22
ELU	90.2	86.7	94.0	85.8	48.0	60.8	65.3	85.0	96.0	90.1	95.1	89.3	89.9	82.80	21
SReLU	91.4	85.6	92.6	83.3	30.0	55.9	69.3	75.0	88.0	82.1	95.7	89.0	89.5	79.02	24
APLU	92.3	87.1	93.2	80.9	25.0	54.1	67.2	76.7	93.0	82.7	95.5	90.3	88.9	78.99	25
GaLU	92.9	88.4	92.2	90.4	41.5	57.8	73.6	89.2	92.7	88.8	94.9	90.3	90.0	83.28	20
sGaLU	92.3	87.9	93.2	91.1	52.0	60.0	72.5	90.0	95.3	87.4	95.4	87.7	88.8	84.13	17
PReLU	92.0	85.4	91.4	81.6	33.5	57.1	68.8	76.3	88.3	82.1	95.7	88.7	89.6	79.26	23
MeLU	91.1	85.4	92.8	84.9	27.5	55.4	68.5	77.1	90.0	79.4	95.3	89.3	87.2	78.76	27
wMeLU	92.9	86.4	91.8	82.9	25.5	56.3	67.5	76.3	91.0	82.5	94.8	89.7	88.8	78.95	26
SReLU(255)	92.3	89.4	93.0	90.7	56.5	59.7	73.3	91.7	98.3	89.0	95.5	89.7	87.9	85.15	13
APLU(255)	95.1	89.2	93.6	90.7	47.5	56.9	75.2	89.2	97.3	87.1	95.7	89.7	89.5	84.35	16
GaLU(255)	92.9	87.2	92.0	91.3	47.5	60.1	74.1	87.9	96.0	86.9	95.6	89.3	87.7	83.73	19
sGaLU(255)	93.5	87.8	95.6	89.8	55.0	63.1	76.0	90.4	95.0	85.3	95.1	89.7	89.8	85.09	14
MeLU(255)	92.9	90.2	95.0	91.8	57.0	59.8	78.4	87.5	97.3	85.1	95.7	89.3	88.3	85.26	11
wMeLU(255)	94.5	89.3	94.2	92.2	54.0	61.9	75.7	89.2	97.0	88.6	95.6	87.7	88.7	85.27	10
RandAct	90.2	90.0	94.2	91.6	54.5	62.0	77.3	90.8	95.7	90.5	95.1	89.0	87.1	85.23	12
RandAct(255)	93.2	88.5	94.4	91.6	51.5	59.1	73.9	88.3	94.0	89.1	95.1	86.7	88.0	84.11	18
FusAct10	93.5	90.7	97.2	92.7	56.0	63.9	77.6	90.8	96.3	91.4	96.4	90.0	90.0	86.67	8
FusAct10(255)	95.1	91.3	96.2	94.2	63.0	64.9	78.7	92.5	97.7	87.6	96.5	89.7	89.8	87.46	6
FusRan10	95.4	91.3	95.8	95.1	63.0	64.2	78.9	93.8	98.7	91.1	96.5	90.3	90.2	88.02	5
FusRan10(255)	96.9	91.2	96.8	96.2	58.5	66.6	79.7	92.5	98.3	91.6	96.6	89.7	91.1	88.13	2
FusRan20	97.5	91.4	96.6	95.8	60.5	65.8	79.7	94.2	99.0	90.5	96.6	89.7	90.7	88.30	1
FusAR20	95.7	90.8	97.0	94.4	61.5	64.1	79.5	93.8	98.3	91.4	96.6	91.0	90.5	88.04	4
FusAR20(255)	96.3	91.2	96.6	95.3	62.0	64.9	79.5	93.8	98.3	90.1	96.6	90.3	90.8	88.12	3
FusAct3(255)	93.9	91.5	94.8	93.1	58.5	63.5	77.6	91.3	98.3	88.0	96.3	89.0	89.4	86.55	9
FusRan3(255)	96.3	90.9	95.6	95.1	54.0	62.9	78.7	92.5	98.7	90.9	96.2	90.0	90.5	87.10	7

**Table 6:** Performance of the proposed approaches in the skin datasets (F1-measure).

	Dataset											Avg	Rank
Method	FV	Prat	MCG	UC	CMQ	SFA	HGR	Sch	VMD	ECU	VT		
ReLU	0.759	0.831	0.872	0.881	0.799	0.946	0.950	0.763	0.592	0.917	0.745	0.823	18
leakyReLU	0.753	0.853	0.876	0.875	0.804	0.944	0.955	0.762	0.606	0.921	0.716	0.824	14
ELU	0.682	0.838	0.870	0.834	0.791	0.941	0.944	0.763	0.540	0.918	0.677	0.800	27
SReLU	0.722	0.839	0.867	0.860	0.807	0.950	0.958	0.743	0.610	0.919	0.709	0.817	25
APLU	0.774	0.840	0.874	0.880	0.796	0.942	0.945	0.761	0.593	0.914	0.745	0.824	16
GaLU	0.759	0.827	0.867	0.872	0.795	0.941	0.933	0.755	0.562	0.913	0.731	0.814	26
sGaLU	0.779	0.834	0.872	0.867	0.798	0.946	0.951	0.766	0.597	0.915	0.739	0.824	15
PReLU	0.785	0.852	0.878	0.886	0.809	0.947	0.953	0.770	0.633	0.924	0.740	0.834	10
MeLU	0.768	0.861	0.878	0.879	0.819	0.947	0.953	0.768	0.643	0.927	0.725	0.834	11
wMeLU	0.768	0.869	0.878	0.888	0.821	0.945	0.956	0.771	0.616	0.929	0.706	0.832	12
SReLU(255)	0.758	0.831	0.872	0.879	0.797	0.946	0.949	0.764	0.592	0.916	0.744	0.823	19
APLU(255)	0.755	0.839	0.873	0.873	0.797	0.940	0.947	0.760	0.584	0.909	0.744	0.820	22
GaLU(255)	0.776	0.832	0.870	0.869	0.790	0.938	0.940	0.758	0.566	0.911	0.756	0.819	24
sGaLU(255)	0.769	0.845	0.876	0.886	0.797	0.944	0.951	0.764	0.617	0.919	0.741	0.828	13
MeLU(255)	0.757	0.836	0.874	0.872	0.792	0.943	0.944	0.767	0.570	0.913	0.744	0.819	23
wMeLU(255)	0.759	0.832	0.873	0.880	0.799	0.946	0.950	0.763	0.599	0.917	0.742	0.824	17
RandAct	0.757	0.852	0.876	0.889	0.804	0.937	0.947	0.764	0.569	0.920	0.730	0.822	20
RandAct(255)	0.732	0.844	0.873	0.878	0.797	0.944	0.937	0.758	0.595	0.914	0.751	0.820	21
FusAct10	0.796	0.864	0.884	0.899	0.821	0.951	0.959	0.776	0.671	0.929	0.748	0.845	3
FusAct10(255)	0.791	0.854	0.881	0.897	0.813	0.949	0.955	0.774	0.654	0.925	0.761	0.841	8
FusRan10	0.795	0.864	0.883	0.901	0.818	0.949	0.958	0.775	0.667	0.927	0.752	0.844	7
FusRan10(255)	0.800	0.867	0.884	0.906	0.819	0.950	0.958	0.779	0.655	0.927	0.749	0.845	5
FusRan20	0.800	0.867	0.884	0.905	0.819	0.950	0.958	0.778	0.663	0.927	0.752	0.846	2
FusAR20	0.799	0.865	0.884	0.901	0.820	0.951	0.959	0.776	0.673	0.929	0.751	0.846	1
FusAR20(255)	0.798	0.862	0.883	0.903	0.817	0.950	0.957	0.777	0.660	0.927	0.758	0.845	6
FusAct3	0.790	0.874	0.884	0.896	0.825	0.951	0.961	0.776	0.669	0.933	0.737	0.845	4
FusRan3	0.783	0.870	0.883	0.902	0.818	0.951	0.959	0.778	0.635	0.930	0.717	0.839	9

**Table 7:** P-value of the comparison among some tested approaches in the medical image classification experiment (< denotes that the method in row wins, ^ denotes that the method in column wins, = denotes that there are were no statistically significant differences).

Classification	ReLU	wMeLU(255)	FusRan3(255)	FusRan10(255)	FusRan20
**ReLu**	---	^0.0046	^0.0210	^0.002	^0.002
**wMeLu(255)**		---	^0.0024	^0.004	^0.002
**FusRan3(255)**			---	^0.004	^0.002
**FusRan10(255)**				---	=0.7148
**FusRan20**					---

**Table 8:** P-value of the comparison among some tested approaches in the skin segmentation experiment.

Skin Segmentation.	ReLU	PReLU	FusAct3	FusAct10	FusAR20
**ReLu**	---	^0.0059	^0.0029	^0.001	^0.001
**preLu**		---	^0.0020	^0.001	^0.001
**FusAct3**			---	=0.9844	=0.6797
**FusAct10**				---	^0.0938
**FusAR20**					---
